# The Role of the Gut Microbiome in Pathogenesis, Biology, and Treatment of Plasma Cell Dyscrasias

**DOI:** 10.3389/fonc.2021.741376

**Published:** 2021-10-01

**Authors:** Marcin Jasiński, Jarosław Biliński, Grzegorz W. Basak

**Affiliations:** ^1^ Department of Hematology, Transplantation and Internal Medicine, Medical University of Warsaw, Warsaw, Poland; ^2^ Human Biome Institute, Gdansk, Poland

**Keywords:** plasma cell dyscrasias, gut microbiome, multiple myeloma, microbiota, short-chain fatty acids

## Abstract

In response to emerging discoveries, questions are mounting as to what factors are responsible for the progression of plasma cell dyscrasias and what determines responsiveness to treatment in individual patients. Recent findings have shown close interaction between the gut microbiota and multiple myeloma cells. For instance, that malignant cells shape the composition of the gut microbiota. We discuss the role of the gut microbiota in (i) the development and progression of plasma cell dyscrasias, and (ii) the response to treatment of multiple myeloma and highlight faecal microbiota transplantation as a procedure that could modify the risk of progression or sensitize refractory malignancy to immunotherapy.

## Introduction – Pathogenesis of Plasma Cell Dyscrasias

Typical genetic alterations in plasma cell dyscrasias are IgH translocations, hyperdiploidy, and cyclin D dysregulation. These are responsible for initiating changes in B-cell postgerminal centres, which result in the transformation of normal cells into benign tumour cells that cause monoclonal gammopathy of undetermined significance (MGUS) (1). This condition is the preclinical stage of multiple myeloma (MM) and occurs in ~3.2% of the population aged over 50 years (2). MGUS is an asymptomatic condition with elevated serum concentration of M protein. Only rarely does it progress to symptomatic MM (1% of patients/year) (3), which can be associated with symptoms that manifest as a result of hypercalcaemia, renal failure, anaemia, and bone lesions. Smouldering MM (SMM) is an asymptomatic, intermediate stage between MGUS and MM, that carries a 10% risk of progression to symptomatic MM per year during the first five years after diagnosis (4). If it is to be possible to screen intensively, perform prophylactic investigations on, and treat in the early stages only those patients who are most at risk of disease progression, accurate prognostic markers of progression of MGUS or SMM to MM are needed.

During the past few years, evidence has emerged that human gut microbiota play an important role in the progression of MM (5–7). The gut microbiota influence the course of MM and the disease shapes the composition of the bacteria in the intestines (6). These interactions, as described below, are based on the strong reliance of MM cells on proinflammatory cytokines [interleukin (IL)-6, tumour necrosis factor (TNF)-α, IL-13] and the ability of bacteria to recycle nitrogen (8).

Recent studies have yielded plenty of information on the differences in microbiota among MM patients and about longitudinal changes acquired during the treatment as well (9). Some recently identified gut microbes are responsible for inducing an inflammatory environment, both within the gut layer and throughout the whole body. These proinflammatory microbes might contribute to the progression of MGUS to MM (5). If they do, the microbiome composition could be used as a prognostic factor for assessing the risk of MGUS transformation or MM progression.

## Gut Microbiota and Immune System in Health and Disease, Specifically Infections

The colonization of the intestine by microbes plays a key role in the maturation of the host’s immune system (10). Current knowledge about crosstalk between gut microbiota and immune cells derives mainly from experiments conducted on germ-free animals (11). For instance, in germ-free mice the population of αβ and γδ intra-epithelial lymphocytes is significantly reduced (12), there is no production of IgA antibodies (13) and Th17 cells are absent (14). One example of a complicated interplay between gut microbiota and immune cells is the following. Polysaccharide A produced by *Bacteroides fragilis* binds to TLR2/TLR1 (Toll-like receptor) heterodimer connected with Dectin-1 (15). Then, the phosphoinositide 3-kinase (PI3K) pathway is activated, glycogen synthase kinase 3β inactivated, which eventually induces cAMP response element-binding protein expression of anti-inflammatory genes (15). Finally, the secretion of polysaccharide A by *Bacteroides fragilis* leads to the differentiation of Treg cells and influences the balance between Th1 and Th2 populations. On the other hand, butyrate produced by the gut microbiota can promote macrophage differentiation from monocytes through histone deacetylase 3 (HDAC3) inhibition that leads to enhanced antimicrobial host defense (16). These are only a few examples of how intricate the crosstalk on the line gut microbiota - immune cells is.

Gut microbiota can also predict responses to therapies administered in oncology. Chaput et al. showed that the presence of *Faecalibacterium* spp. increases the efficacy of anti-CTLA-4 immunotherapy while probably the *Bacteroides* spp. is associated with inferior responses in metastatic melanoma (17). Moreover, it is recently hypothesized that gut microbiota composition can influence the responses to the CAR-T therapy (18), and bearing in mind recent papers about the efficacy of such therapy in multiple myeloma the discussion about gut microbiota as a predictive marker of response is warranted (19).

The impact of the interplay between the immune system and gut microbiota in the context of infections cannot be forgotten as patients with multiple myeloma are far more prone to infections than the healthy population (20). The ability of microbes to release signaling molecules into the bloodstream can modulate the host’s response to infections *via* the regulation of immune cell development (21). For instance, butyrate secreted by bacteria promotes the differentiation of monocytes in the bone marrow to a tolerogenic phenotype (22). Moreover, it was recently showed that some bacterial species could decrease the level of corticosterone in the blood which could improve the function of the immune system during the infection (23).

## Gut Microbiota and Tumourigenesis

**Figure 1:**
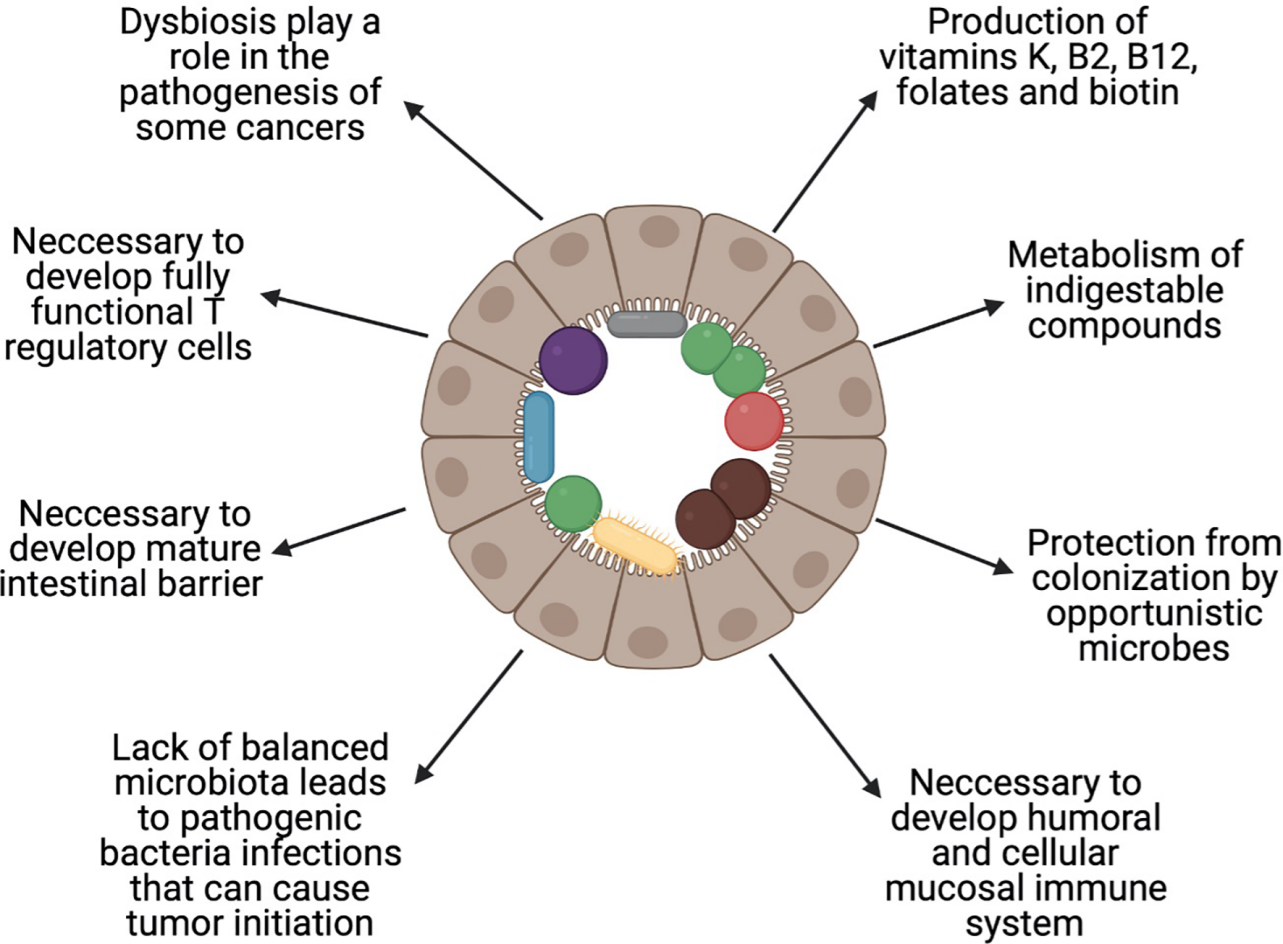
Role of the balanced gut microbiota. Healthy gut microbiota are important in maintaining health. The figure shows the most important roles that are played by the human gut microbiota. Created with BioRender.com.

The available data show that the gut microbiota are more numerous than genes, cells, and enzymatic reactions in the host organism, which suggest their importance for its health. In healthy persons, microorganisms are responsible for production of vitamins K, B_2_ (riboflavin), B_12_ (cobalamin), folates, and biotin (24), metabolism of indigestible compounds, and protection from colonisation by opportunistic bacteria (25), and are necessary for the development of the humoral and cellular mucosal immune systems (26) (**Figure 1**). Along with these advantages of the gut microbiome, there are also some disadvantages. It is well established that dysbiosis, which is an imbalance in the proportion of microbes compared to a healthy state, plays a role in the pathogenesis of colorectal cancer (CRC) (27). Wang et al. showed that there is a difference in the composition of gut microbiota between patients with CRC and healthy individuals (28). A similar influence of microbial dysbiosis, *via* proinflammatory microbe-associated molecular patterns (MAMPs) and bacterial metabolites, has been shown in liver (29) and pancreatic (30) cancer.

The gut microbiota are accompanied by gut-associated lymphoid tissue (GALT), which is the largest peripheral immune organ (31). As many as 60–70% of peripheral lymphocytes are localised within the gut mucosa, so it is not surprising that the number of interactions between immune cells and the gut microbiota is high (32). There are numerous examples of how the gut microbiota and immune system influence each other within the gut mucosa. Brandsma et al. showed that the transplantation of proinflammatory faecal microbiota from *Casp1* ^−/−^ mice to *Ldlr* ^−/−^ mice resulted in systemic inflammation and promoted atherogenesis (33). In contrast, Mason et al. reported that reduced anti-inflammatory gut microbiota was correlated positively with depression. This correlation could be explained by inflammation playing a role in the pathogenesis of depression (34). The crosstalk from microbes to immune cells can be forwarded directly through their metabolites used as messengers, such as MAMPs or damage-associated molecular patterns (DAMPs), or through activation of Toll-like receptors (TLRs) that in turn cause the activation of immune cells (35, 36). Some metabolites, such as short-chain fatty acids (SCFAs), can directly promote the generation of T regulatory (Treg) cells (37) or are responsible for transforming growth factor-β production in epithelial cells within the gut. This in turn promotes Treg-cell confluence in the gut mucosa, which inhibits the activation of immune cells (38). Germ-free (GF) mice that are deprived completely of gut microbiota comprise excellent examples of the importance of gut bacteria for efficient immune function (26). In GF mice, Treg cell function is impaired, which suggests that gut microflora are necessary for the development of a fully functional Treg cell population (39). In GF mice, the intestinal barrier is immature, which results in increased mucosal permeability (40). This is a key mechanism that leads to the development of inflammatory bowel disease or enteric infections (40). Colonisation of GF animals with normal gut microbiota leads to increased systemic immunological capacity, different patterns of migration of immune cells, significant changes in the production of specific antibodies, a general increase of immunoglobulin production, and changes in mucosal-associated lymphocyte tissues and cell populations (41–43).

In summary, in general, the micro-organisms in the gut are beneficial, but under certain conditions can have a damaging effect, in severe cases promoting the growth of cancer cells.

## Comparison of the Gut Microbiome in Patients With Plasma Cell Dyscrasias and Healthy Individuals

In recent years, scientists have confirmed the link between certain kinds of tumours and the composition of gut microbiota. For example, in CRC, many changes in the composition of bacterial species that colonise the gut have been identified and their contribution to tumourigenesis confirmed. Specific bacterial species colonizing the gut have even been indicated as possible markers of early diagnosis of CRC (44).

Regarding plasma cell dyscrasias and the gut microbiome, recent evidence shows metagenomic changes in the composition of commensal bacteria and frequent colonisation by opportunistic bacteria. Jian et al. performed a study on samples collected from 19 patients who had been newly diagnosed with MM and 18 healthy controls (6). They observed significant differences in the composition of bacteria in the gut between these two groups. One of the main changes was the increase of nitrogen-recycling bacteria, such as *Klebsiella* and *Streptococcus*, which are opportunistic pathogens that are responsible for infections associated with high mortality in this immunocompromised population. It has been suggested that this change might be due to the high serum concentration of urea in patients with MM, which results from increased production of NH_4_^+^ by tumour cells and restricted secretion of urea due to impaired renal function (45). The mechanism presented above is responsible attracting nitrogen-recycling bacteria to the gut. Changes in diversity in gut microbiota have been reported, which indicates that samples from MM patients are characterised by increased diversity and poorer interactions between genera (6), although other studies have produced results that indicate contrary phenomena (46, 47). Furthermore, samples from MM patients included a reduced number of SCFA-producing bacteria, which affect tumourigenesis in plasma cell dyscrasias (see below) (6). Other changes in the composition of commensal bacteria, and colonisation with opportunistic pathogens, occur because of the treatment of MM. Unfortunately, research in this field is limited to the study of bacterial composition only. Further research, which studies differences in the balance and numbers, etc., of fungi, viruses, and eukaryotic organisms are needed (**Table 1**).

**Table 1:** Summary of the alterations of the gut microbiota in MM patients.

Gut microbiota of MM patients
Frequently colonised with opportunistic bacteria (6)
Increase in the number of bacteria involved in nitrogen recycling, such as *K. pneumoniae* or *S, pneumoniae* (6)
Increased diversity and poorer interactions between genera (6)
Reduced number of SCFA-producing bacteria (6)
Changes resulting from applied treatments especially antibiotics

## Influence of the Gut Microbiome on the Development and Progression of Plasma Cell Dyscrasias

As mentioned previously, MGUS is an asymptomatic state that occurs in ~3.2% of people aged over 50 (1). Only a small percentage of patients progress to symptomatic MM. For many years, researchers have wanted to identify the factors responsible for the development of plasma cell dyscrasias, and the reasons why some patients progress to MM whereas others do not.

Researchers have shown that there are no significant genetic differences between MGUS and MM cells. This suggests that environmental conditions could be an important factor in determining the risk for progression, although such factors are not necessarily present at the time at which MGUS develops. Therefore, tumour microenvironment seems to be a strong predictor of MGUS progression. Given the high degree of heterogeneity between clones in plasma cell dyscrasias, it is probable that only clones that are developing in a favourable niche will become an initiation point for further progression. As mentioned previously, proinflammatory TME in the bone marrow is needed for successful progression from MGUS to symptomatic MM, but it is a further issue how the gut microbiota can influence this microenvironment and contribute to tumour progression.

### Short-Chain Fatty Acids

SCFAs are bacterial products that are responsible for ion absorption, gut motility, and modulation of immune responses (48). SCFAs can inhibit the nuclear factor kappa-light-chain enhancer of activated B cells (NF-κB) and such proinflammatory cytokines as IL-6 and TNF-α which are playing the role in activating osteoclasts to create niches for myeloma cells and additionally promote differentiation of Th17 cell (49). In contrast, SCFAs may also increase the level of IL-10 and induce expression of FoxP3 which in turn leads to differentiation of immunosuppressive CD4^+^ T cell subset (Treg) (48). Eventually, both Treg (IL-10 and TGF-β) and Th17 (IL-17) cells secrete cytokines that promote MM cell proliferation *via* positive feedback loop (50). One SCFA, butyrate, is reported to increase T-cell apoptosis by HDAC-dependent Fas upregulation and consequent Fas-mediated apoptosis of T cells. That in turn inhibit T-cell accumulation within inflamed colonic mucosa which could prevent antigenic stimulation known for its role in multiple myeloma development (51). Furthermore, Jian et al. showed that SCFA-producing bacteria such as *Anaerostipes hadrus, Clostridium butyricum, and Clostridium saccharobutylicum* were reduced in patients with MM, and that the addition of *Clostridium butyricum* in a mouse model of MM resulted in mitigation of tumour progression (6). SCFAs are also involved in the response to treatment. Small, uncontrolled studies have indicated that SCFA-producing bacteria play a significant role in reducing the level of proinflammatory cytokines, thereby protecting the host from tumour progression. Loss of SCFA-producing bacteria can result in a higher risk of tumour progression. Bearing in mind that specific diets can increase the population of SCFA-producing bacteria, studies are needed to investigate whether changes in diet in patients with MGUS can influence the risk of tumour progression.

### L-Glutamine

Jian et al. showed that stool samples from MM patients had higher concentrations than in healthy patients of bacteria that are involved in nitrogen utilisation and recycling, such as *Klebsiella* and *Streptococcus* (6). The following mechanism has been proposed to explain this phenomenon (6). MM cells are known producers of NH_4_^+^ (52), which results from uptake of glutamine (53). This NH_4_^+^ then accumulates in the bone marrow and is released into the blood. In a healthy organism, the liver successfully converts NH_4_^+^ into urea in the urea cycle. However, MM patients experience a high increase in blood NH_4_^+^ level that exceeds the capacity of the liver to convert it to urea and can even result occasionally in hyperammonaemic encephalopathy (54). In addition, monoclonal protein renal deposition and consequent reduction in renal function mean that the process of urea excretion is impaired severely (55). Taken together, these factors lead to an increased concentration of urea in the blood, such that excessive amounts of urea reach the intestinal lumen. The presence of urea in the gut layer causes the selection of nitrogen-recycling bacteria, such as *Klebsiella* and *Streptococcus.* These bacteria are involved in the hydrolysis of urea and synthesis of L-glutamine that is taken up by MM cells, which promotes tumour progression. It is probable that MM cells harness the gut microbiota of the host as a recycler of NH4^+^ to deliver the necessary L-glutamine. In light of this, we speculate that targeting human microbiota with natural methods, or antibiotics, if necessary, could be an attractive strategy to stop this vicious cycle.

### Th17 Cells

The differentiation of Th17 in GF mice is inhibited (14). Microbial colonization, especially with segmented filamentous bacteria (SFB) promotes induction of Th17 cells (56). Furthermore, it is already known that Th17 elicited by SFB are of non-inflammatory phenotype while Th17 cells induced by other bacteria *Citrobacter* are secreting plenty of proinflammatory cytokines (57).

Plasma cells express IL-17 receptors on their surface and are stimulated *in vitro* and *in vivo via* IL-17 produced by Th17 cells (58). Of note, IL-6-STAT3 signalling pathway activated by IL-17 is relevant both for tumour (59) and plasma cell (60) growth which suggests the role of IL-17 during different stages of MM. IL-17 causes the upregulation of the receptor activator of the NF-κB ligand, which results in the activation of osteoclasts (61) and eosinophils that are producing IL-6 and TNF-α (5). Hence, IL-17 is the cytokine that bears the principal responsibility for bone lesions in plasma cell dyscrasias. Stromal cells respond to IL-17 as well by producing IL-6 (62). Moreover, the interplay between IL-6 and TGF-β, that are highly expressed in the bone marrow of patients with MM, is influencing the generation of Th17 cells (49).

*Prevotella heparinolytica* is responsible for the differentiation of Th17 cells and their migration to the bone marrow in the Vk*MYC mouse model of MM (5). In mice that lacked IL-17, the progression of plasma cell dyscrasias was delayed. Inhibition of IL-17, IL-17 receptor A, and IL-5 in a Vk*MYC model with monoclonal antibodies results in reduced accumulation of Th17 cells and eosinophils in the bone marrow, which results in delayed tumour progression (5).

**Figure 2:**
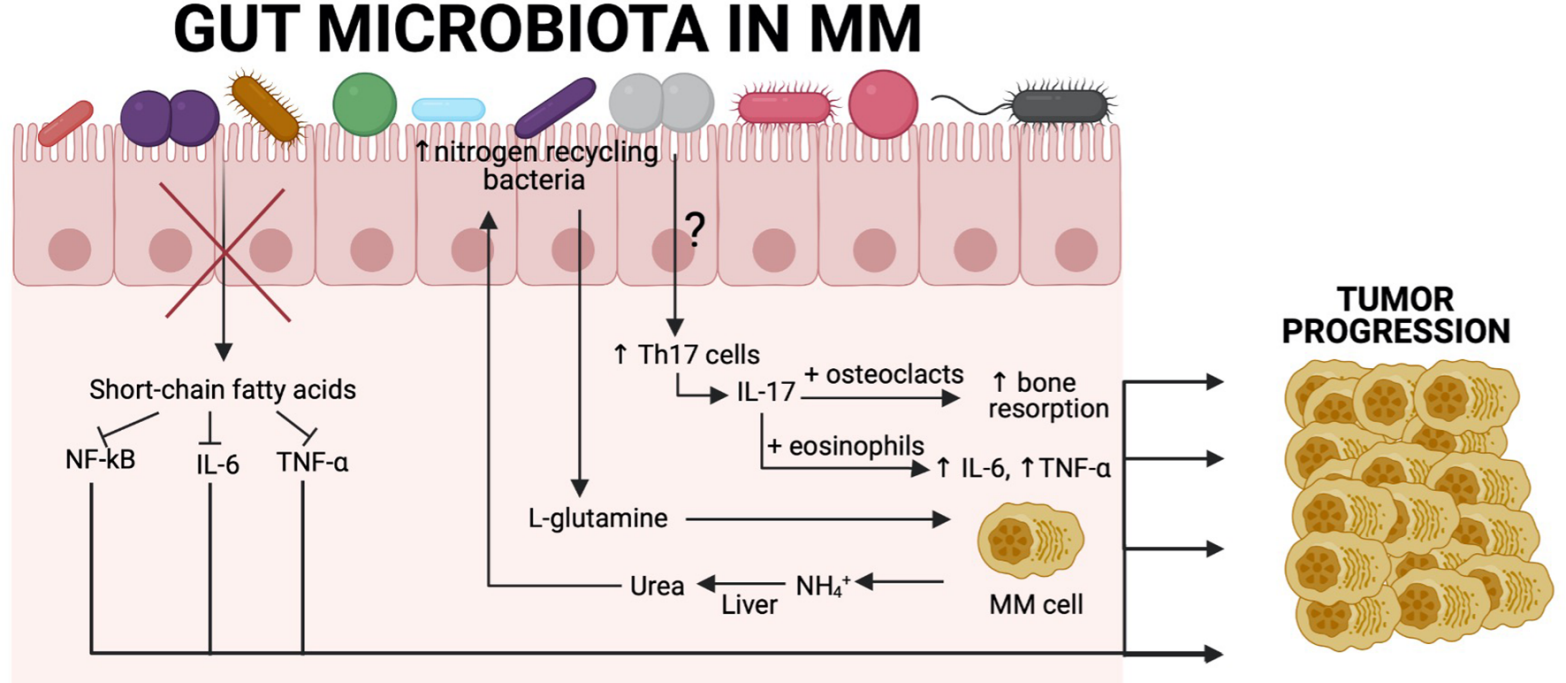
Association between the gut microbiota and tumour progression in MM patients. Recent findings show a close relationship between gut commensal microbiota and MM cells. SCFA-producing bacteria are significantly reduced, resulting in increased levels of NF-κB, IL-6, and TNF-α, which are known to contribute to tumor progression in MM. Another example derives from the fact that MM patients have increased nitrogen-recycling bacteria. These bacteria are involved in L-glutamine production, which is an essential amino acid for MM cells. MM cells produce high amounts of NH4^+^, which is transformed in the liver into urea and reaches high concentrations in the blood and can select nitrogen-recycling bacteria such as *K. pneumoniae* or *S. pneumoniae.* The influence of the gut microbiota on Th17 cell differentiation in MM patients remains to be characterised, although we know that in a Vk*MYC mouse model, *P. heparinolytica* was responsible for that. Patients with MM have significantly higher level of IL-17 in the blood, which is produced by Th17 cells and causes bone resorption, resulting in bone lesions that are the main symptom of this malignancy. Additionally, IL-17 activates eosinophils that are consequently producing proinflammatory cytokines (IL-6 and TNF-α) that are involved in tumor progression. Created with BioRender.com.

Patients with MM have elevated serum level of IL-17 but interestingly after therapy with bis-phosphonate level of that cytokine is reduced (63). A higher level of IL-17 is also seen in the blood of patients with SMM and is a predictor of rapid progression of tumour growth. Therefore the level of IL-17 could be used as a potential marker of high-risk SMM patients (64). Similar to the Vk*MYC model, it would be useful to initiate studies on patients to determine which bacteria are involved in Th17 differentiation. Using this approach, bacteria that are involved indirectly in the development of bone lytic lesions, which is one of the main causes of morbidity in MM patients, could be eradicated (**Figure 2**).

## The Link Between the Gut Microbiome and Treatment in Plasma Cell Dyscrasias

It is known that different results of treatment and toxicity profiles are associated with the gut microbiome (65, 66). For instance, a specific composition of gut microbiota is required for an optimal response to treatment with immune checkpoint inhibitors (67). Baruch et al. conducted a phase I study on faecal microbiota transplantation from complete responders to treatment for metastatic melanoma to 10 non-responders, which resulted in partial responses in three patients and a complete response in one (68). The gut microbiome can influence the results of treatment, especially in respect of adverse events, and treatment can modulate the gut microbiome.

During the last decade, new treatments for plasma cell dyscrasias have been introduced, including immunomodulatory drugs (thalidomide, lenalidomide, and pomalidomide), proteasome inhibitors, and monoclonal antibodies. These have improved the length and quality of life of patients with MM (69). To emphasise the role of the gut microbiome in plasma cell dyscrasias, we describe how microbes can affect the outcomes of treatment in plasma cell malignancies. Their role is particularly visible in respect of possible infectious complications after treatment that are due to infection. It was recently confirmed that treatment of MM changes the composition of the gut microbiome in respect of diversity (70).

Pianko et al. showed that MM patients with no minimal residual disease (MRD) after completion of upfront therapy had greater numbers of butyrate-producing *Eubacterium halii* than MRD-positive patients (71). Similarly, another butyrate producer, *Faecalibacterium prausnitzii*, was associated with an absence of MRD (71). Moreover, Peled et al. showed that intestinal *Eubacterium limosum* was associated with decreased risk of MM relapse after allogeneic haematopoietic cell transplantation (72). These observations suggest that changes in commensal microbiota caused by MM treatment could influence the entire process of therapy or be a predictor of a better response. Gopalakrishnan et al. showed how significant the impact of the changes in the gut microflora on the response to treatment can be. They showed that melanoma patients who responded well to immunotherapy with anti-PD-1 agents had a relative abundance of *Ruminococcaceae* family and higher alpha diversity (diversity within one sample) in faecal microbiome samples (73). Thus, it is possible that the composition of gut microbiota in MM patients has a major influence on the outcomes of immunotherapy, especially taking into account that MM, similarly to melanoma, is closely related to immune response.

### Proteasome Inhibitors

PIs, such as bortezomib or carfilzomib are commonly used in primary and relapsed MM. One common adverse effect is gastrointestinal (GI) toxicity that results in diarrhoea. First, it was thought that PIs alter gut motility or cause neurotoxicity, resulting in autonomic neuropathy. The molecular reason for GI toxicity is now established as an increase in TNF-α receptor 1 expression on intestinal cells and higher concentrations of IL-6, TNF-α and IL-1β (74). However, there is a lack of evidence that PIs influence composition of the gut microbiota. It might be that inhibition of the NF-κB pathway is responsible for GI toxicity of PIs (75). SCFAs can suppress the NF-κB pathway, which could augment GI toxicity of PIs (76).

### Steroids

Steroids are among the most commonly used anti-inflammatory drugs. They are used in chemotherapy regimens for MM, as well as in the treatment of a wide range of rheumatoid diseases. Huang et al. showed that mice that had been subjected to chronic exposure to steroids differed in the composition of their gut microbiota compared with their healthy counterparts (77). Steroid-treated mice had an increase in *Bifidobacterium* and *Lactobacillus*, which are both associated with anti-inflammatory effects, whereas they noted an absence of *Mucospirillum*, which is responsible for degradation of colonic mucin. This effect might be explained by the decrease of mucin production in mice treated chronically with steroids. Dexamethasone exerts its anti-inflammatory effects by blocking the NF-κB pathway (78). Furthermore, mice that were treated with dexamethasone produced less IL-17 than healthy mice (77). This may be another case in which steroids reshape the intestinal flora, since IL-17 production depends on Th17 cell differentiation, which is associated with specific gut microbiota. However, not only chronic exposure to, but also acute treatment with, steroids affected gut microbiota in mice (77). Ünsal et al. showed that rodents that were injected with a single, strong dose of dexamethasone underwent an increase in the number of ileal anaerobic bacteria. Moreover, a single injection of a low dose of dexamethasone resulted in an increase in the population of coliform bacteria (79). However, the long-term effect of these changes remains to be determined.

### Antimicrobials

The link between antibiotics and the gut microbiome seems to be the most examined and the influence of this group of drugs on commensal bacteria is well established. However, although this link has been studied intensively in healthy volunteers, there remains a lack of wider studies with many groups of antibiotics in MM patients. Ziegler et al. showed that levofloxacin, which is the most commonly prescribed drug for bloodstream infections and neutropenic fever prophylaxis, had a less damaging effect on intestinal microbiota than broad-spectrum β-lactam (BSBL) antibiotics (80). The latter group reduced alpha diversity. The former was not associated with specific changes in the gut microbiome that had been found to be associated with poor clinical results (decrease in populations responsible for protection against *C. difficile*; increase in non-Bacteroidetes taxa, and reduction of alpha diversity). In light of their results, the authors emphasised that fluoroquinolone antibiotics protected patients from the negative effects of BSBLs (80). In MM patients who had been newly diagnosed and who were at particular risk of infection, the effect of prophylactic antibiotics was small and there was no decrease in early mortality (81). However, Valkovic et al. reported that MM diagnosis or progression was frequently preceded by infection (82). That could have been because bacterial infections are associated with robust production of proinflammatory cytokines and TLR activation on MM cells (83, 84). This is why prophylactic broad-spectrum antibiotics can result in a delay in disease progression. In respect of allogeneic stem-cell transplantation (alloSCT), Weber et al. showed that early use of broad-spectrum antibiotics that are active against commensal organisms, such as *Clostridiales* was associated with increased transplant-related mortality and decreased overall survival (85). Administration of imipenem–cilastatin or piperacillin–tazobactam for neutropenic fever resulted in gut microbial perturbation and increased graft-*versus*-host disease-related mortality compared with aztreonam or cefepime, both of which decreased activity against commensal, anaerobic bacteria (86). Such observations of antibiotic effects on the response to treatment of MM need to be investigated in patients who are treated with autologous stem-cell transplantation (ASCT). There is also a recently published systematic review of infections associated with selinexor in patients with relapsed/refractory MM that also compares the risk of infections with other novel agents. It is already known that selinexor could prevent viral infections through blocking of XPO1 - mediated nuclear transport which facilitates the export of viral proteins. The authors state that randomized clinical trials are needed to fully understand the risk of infections associated with selinexor (87).

### Autologous Stem-Cell Transplantation

D’Angelo showed that after ASCT, patients showed significantly decreased diversity of the microbial gut population (88). El Jurdi et al. showed an association between baseline microbiota of patients undergoing ASCT with further regimen-related toxicities and with the rate of neutrophil engraftment (89). They found that bacterial diversity after ASCT recovered within 1 month after the procedure, but that fungal populations constantly decreased, which suggests that a longer time is needed for the reconstitution of the mycobiome. Although the prospective study included only 15 patients, the results were encouraging for further studies. This group recognised several links between the composition of the microbiota and effects on ASCT-related toxicity and outcomes. One of the links relied on identifying an increased population of *Bacteroides* at day +7 in patients with less severe diarrhoea, while more severe diarrhoea, nausea, and vomiting occurred in patients with a higher prevalence of the stool populations of *Blautia* and *Ruminococcus*. They also identified a negative correlation between fungal phyla *Glomerella* presence in stools and neutrophil engraftment (89). Similar conclusions were drawn from the results of the small pilot study with 15 patients, showing that baseline microbiota were associated with subsequent incidence and severity of nausea, vomiting, neutropenic fever, and rate of neutrophil engraftment (90). Khan et al. showed recently that 534 adult recipients of high-dose chemotherapy with ASCT had significantly decreased alpha diversity at early pretransplant stages than healthy individuals and that this reduction in diversity tended to be more marked in the course of the procedure (9). The pattern of this loss of diversity and dominance of specific taxa were similar to those seen in patients after alloSCT. In addition, they showed that the greater the diversity of the gut microbiota, the lower risk of progression or death. Our group showed in a retrospective, single-centre study that colonisation with antibiotic-resistant bacteria had a significant influence on the outcomes of alloSCT (91). The main finding was that the overall survival of patients who were colonised by antibiotic-resistant bacteria was estimated to be half that of the noncolonised group. A similar conclusion was reached by Scheich et al. concerning the effect of colonisation by multidrug-resistant organisms on the results of ASCT (92).

### Other Treatments

There is little information on the possible influence of other treatments, such as immunomodulatory drugs and monoclonal antibodies, on plasma cell dyscrasias (**Table 2**).

**Table 2:** Relationship between the gut microbiota and treatment of plasma cell dyscrasias.

Treatment	How it affects the gut microbiota in plasma cell dyscrasias?
PIs	There is no evidence proving the influence of PIs on gut microbiota
Steroids	Mice treated with steroids had increased *Bifidobacterium* and *Lactobacillus* population and the absence of *Mucospirillum* bacteria (77) Mice treated with dexamethasone had decreased production of IL-17 compared with an untreated group. IL-17 production is strictly related to the presence of Th17 cells, whose differentiation in the gut was recently proved in the Vk*MYC mouse model. This indicates some relationship (77) Not only chronic exposure but also acute treatment resulted in alteration of the gut microbiota in rodents (79)
Antimicrobials	Levofloxacin had no significant impact on the human gut microbiota, while BSBL antibiotics caused a reduction of alpha diversity (80) Administration of broad-spectrum antibiotics efficient against commensal microbiota resulted in higher transplant-related mortality and decreased overall survival (85) Patients treated with imipenem–cilastatin or piperacillin–tazobactam had increased risk of GVHD-related mortality compared with aztreonam or cefepime (86)
ASCT	Patients after ASCT had decreased diversity of microbial populations in the gut and the normal composition was rebuilt within 1 month after the procedure (89) There is a strong relationship between baseline microbiota of MM patients and severity of toxicity related to the procedure and with the rate of neutrophil engraftment (89) Patients after high-dose chemotherapy before ASCT had significantly decreased alpha diversity of the gut microbiota compared with healthy individuals (9)
Other treatments	Little is known about possible influence of gut microbiome on treatment outcomes with immunomodulatory drugs or monoclonal antibodies

## Conclusions

Despite some progress in the outcomes of treatment of MM, it remains a disease that cannot currently be cured, due to relapse or refractoriness to any available therapy. An emerging factor that could influence not only the refractoriness of MM but also a progression from asymptomatic MGUS to MM is the gut microbiota. We see that changes in the composition of commensal bacteria can affect the process of transforming MGUS to MM. Further, these changes are associated with colonisation with opportunistic pathogens that can become an aetiological agent of complications due to infection that are associated with treatment. Probably, in the future, it will be possible to identify patients who have an especially high risk of progression to MM, or even to modulate intestinal microflora to reduce the risk of progression of MGUS. It is also possible that the gut microbiota will be modulated to reduce complications that are due to treatment and disease, or to improve treatment outcomes. However, the field of microbiota in MM is still in its infancy and further work is required to gain a fuller understanding of the phenomena.

## Author Contributions

All authors contributed to the article and approved the submitted version.

## Conflict of Interest

JB and GWB are the founders of the faecal microbiota bank and laboratory named the Human Biome Institute.

The remaining authors declare that the research was conducted in the absence of any commercial or financial relationships that could be construed as a potential conflict of interest.

## Publisher’s Note

All claims expressed in this article are solely those of the authors and do not necessarily represent those of their affiliated organizations, or those of the publisher, the editors and the reviewers. Any product that may be evaluated in this article, or claim that may be made by its manufacturer, is not guaranteed or endorsed by the publisher.
